# Short-Term Outcomes of Polycarbophil and *Propionibacterium acnes* Lysate Gel after Open Hemorrhoidectomy: A Prospective Cohort Study

**DOI:** 10.3390/jcm9123996

**Published:** 2020-12-10

**Authors:** Gaetano Gallo, Ugo Grossi, Gian Luca Di Tanna, Giulio Aniello Santoro, Gilda De Paola, Giuseppe Clerico, Alberto Realis Luc, Mario Trompetto, Giuseppe Sammarco

**Affiliations:** 1 Department of Medical and Surgical Sciences, University of Catanzaro, Viale Europa, 88100 Catanzaro, Italy; gilda_depaola@libero.it; 2 Tertiary Referral Pelvic Floor Center, IV Surgery Unit, Treviso Regional Hospital, DISCOG, University of Padua, 31100 Treviso, Italy; grossiugo@gmail.com (U.G.); giulioasantoro@yahoo.com (G.A.S.); 3 Statistics Division, The George Institute for Global Health, Faculty of Medicine, University of New South Wales, Sidney, NSW 2042, Australia; glditanna@gmail.com; 4 Department of Colorectal Surgery, S. Rita Clinic, 13100 Vercelli, Italy; clerico.giuseppe@gmail.com (G.C.); alberto.realisluc@libero.it (A.R.L.); trompetto.mario@libero.it (M.T.); 5 Department of Health Sciences, University of Catanzaro, Viale Europa, 88100 Catanzaro, Italy; sammarco@unicz.it

**Keywords:** Emorsan^®^Gel, hemorrhoidal disease, hemorrhoidectomy, pain, wound healing

## Abstract

Background: Pain is the most common complication after open excisional hemorrhoidectomy (OEH). We assessed the effectiveness of polycarbophil and *Propionibacterium acnes* lysate gel (Emorsan^®^Gel) on pain control after OEH. Research design and methods: Fifty consecutive patients undergoing OEH were included. All patients received stool softeners and oral analgesia in the post-operative period. Emorsan^®^Gel was also used topically by the last 25 patients (Emorsan^®^Gel group (EG)) until Post-Operative Day 20 (POD 20). The primary outcome was the effectiveness of Emorsan^®^Gel on pain relief using an 11-point visual analogue scale (VAS). Morbidity, wound healing (WH), and time to work were documented at POD 1, POD 10, POD 20, and POD 40. Results: Of the 50 patients enrolled, twenty-eight (56%) were males; median age, 49 (range, 28–73) years. The VAS score decreased over time in all patients, with significantly lower scores at POD 20 in the EG (1.44 (SD, 1.16) vs. 2.12 (0.93) in the control group (CG); *p* = 0.045). All patients in the EG achieved complete WH at last follow-up, compared to only 17 (68%) in the CG (*p* = 0.004). The likelihood of WH was 66% higher in the EG (OR, 1.66 [95%CI, 0.80–3.44; *p* = 0.172). Conclusions: Emorsan^®^Gel is safe and effective at reducing pain after EOH, promoting earlier WH compared to standard care treatment.

## 1. Introduction

Open excisional hemorrhoidectomy is the gold standard treatment of III and IV degree hemorrhoidal disease (HD) [1]. However, despite technological advances (e.g., radiofrequency and ultrasound devices) [2,3], the post-operative period remains a very delicate phase that can deeply affect patient’s quality of life. Bleeding, pain, and anal stricture are among the complications of hemorrhoidectomy, which may require re-intervention in the short or long term.

Post-operative pain represents a major burden for patients. It is frequently experienced during the first 7–10 days after surgery [4] and may arise from the incorporation of sensitive anal mucosa or fibers of the internal anal sphincter into stitches, delayed wound healing, hard stool consistency, or edema of the mucocutaneous bridges. Technical advice allied with the optimization of post-operative analgesia may help prevent some of these triggers [5,6]. In a single-blind randomized trial comparing pedicle coagulation vs. ligation during excisional hemorrhoidectomy, a better control of post-operative pain was observed after pedicle coagulation, demonstrated by a reduced number of required analgesics [7].

Emorsan^®^Gel (Depofarma S.p.A, Treviso, Italy) is a topical gel rich in dimethicone and cyclopentasiloxane. These substances protect the skin and mucous membranes from external agents [8,9]. Their property of forming occlusive barriers on the epidermis helps to reduce inflammation and itching, while promoting the healing process [10]. Furthermore, lactic acid can restore the local pH to physiological levels, with a positive impact on wound healing [11].

The aim of the present study is to assess the short-term outcomes of Emorsan^®^Gel in the post-operative management after open diathermy excisional hemorrhoidectomy.

## 2. Patients and Methods

Between January and December 2018, fifty consecutive patients were prospectively included in the “EMORGEL Study”, approved by regional ethics committee “Sezione Area Centro, Regione Calabria”; Approval Code 84/19). Written informed consent was obtained from all patients.

Consecutive subjects aged between 18 and 75 years, undergoing open diathermy excisional hemorrhoidectomy for Goligher III or IV degree HD were included in this study. The procedures were performed according to the PROSPECT (PROcedure-SPECific post-operative pain management) evidence [12], and neither ligation of the vascular pedicle, nor concomitant internal sphincterotomy were applied [13].

Pre-specified exclusion criteria were: pregnancy, current use of specific medications (e.g., psychopharmaceuticals, antibiotics, antimycotics, immunomodulators, corticosteroids, or other immunosuppressive drugs), active cancer, previous open hemorrhoidectomy, known allergy to product components.

A block enrolment strategy was used with two cohorts of 25 patients (control group (CG) and Emorsan^®^Gel group (EG)) enrolled at intervals of 6 months (from January to June and from July to December 2018, respectively). All patients received standard care treatment in the immediate post-operative period, consisting of stool softeners and oral analgesia (a recommended oral dose of ketorolac tromethamine of 10 mg every 4–6 h as needed, not exceeding 40 mg per day for 5 consecutive days, according to the short-term management of moderate/severe acute post-operative pain). Only the last 25 patients enrolled (i.e., in the second half of 2018) also received Emorsan^®^Gel topically every 12 h (2 mL per dose) for 20 consecutive days.

All patients were followed up at 4 time points: T1, Post-Operative Day 1 (POD 1); T2, POD 10; T3, POD 20; T4, POD 40. The following data were recorded on each visit: pain severity (spontaneous and/or on defecation) using an 11-point visual analogue scale (VAS); presence of thrombosis (defined as one or more swollen painful piles at the site of the mucocutaneous bridge); hemorrhage; wound status (granulating; healed); use of analgesia; grade of satisfaction on a 5-point scale (insufficient; sufficient; more than sufficient; good; excellent). Data on bowel habit were also recorded at T2, T3, and T4 using three patterns of the Bristol stool scale (hard, 1–2; normal, 3–5; non-formed, 6–7) [14]. During the same appointments, patients were asked to report the grade of activities they could sustain using a 4-item scale: complete inactivity, total autonomy at home, ability to drive, or return to normal activities (i.e., autonomy at home, driving, and working).

Any adverse events related to the use of Emorsan^®^Gel were recorded at T2, T3, and T4 from the last 25 patients enrolled, using a 6-point score: 0, no erythema; 1, very mild erythema; 2, moderate erythema without edema; 3, moderate erythema with edema without papules; 4, severe erythema with edema +/− papules; 5, severe erythema with edema and vesicles.

Primary outcome was the effectiveness of Emorsan^®^Gel on pain relief. Secondary outcomes were post-operative morbidity, wound healing, and time to work and social life.

All data were recorded anonymously on a prospectively built electronic database.

The study is reported in accordance to the STROBE (Strengthening the Reporting of Observational Studies in Epidemiology) guidelines [15].

### Statistical Analysis

Continuous variables are presented as the mean and standard deviation, while binary variables as proportions. Comparisons across groups were made using ANOVA and Fisher’s exact tests, respectively. *p*-values were reported at their nominal value. Uni- and multi-variable logistic regressions were performed with a pre-defined covariate set, which included age and gender. All statistical analyses were performed using Stata 16 (College Station, TX: StataCorp LLC, Texas, TX, USA).

## 3. Results

Of the 50 patients enrolled, twenty-eight (56%) were males. Median age was 49 (interquartile range (IQR), 43.5–56) years. Gender (males, 60% vs. 52%, respectively; *p* = 0.776) and age (median, 51 vs. 48, respectively; *p* = 0.899) were similarly distributed in the two groups (Table 1).

Mean operative time was 24.3 (SD, standard deviation 6.4; range 17–37) minutes in the EG and 25.3 (SD, 6.5; range 18–39) minutes in the CG. Morbidity occurred in six (12%) patients: bleeding (*n* = 4, requiring reintervention in two patients, one per group) and urinary retention (*n* = 2, one per group).

Daily use of analgesia up to POD 5 was also similar, with a significant drop after POD 1.

**Figure 1:**
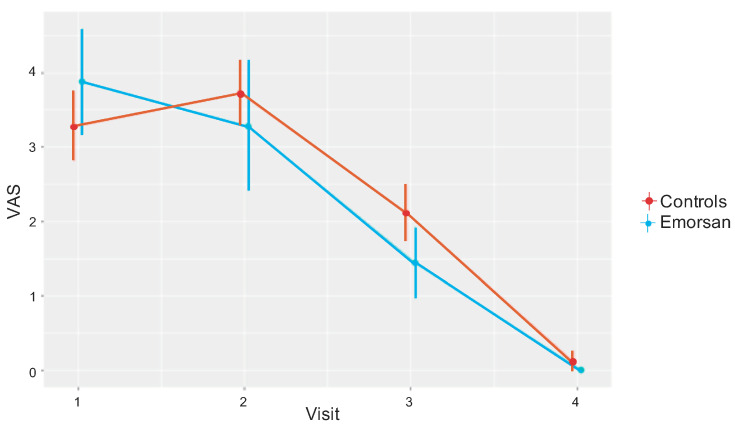
Visual analogue scale (VAS) mean scores in the two groups at each follow up visit.

The VAS score decreased likewise over time (Figure 1). However, patients in the EG had a significantly lower mean score at POD 20 (1.44 (standard deviation, 1.16), compared to 2.12 (0.93) in the CG; *p* = 0.045). Although not reaching statistical difference, the VAS mean score over time was 0.1 points lower in the EG vs. CG (Table 2).

Thrombosis was observed at T2 and T3 in two (8%) patients in the CG and spontaneously resolved at T4. None of the patients in the EG experienced this complication, nor adverse events. No cases of hemorrhage were noted.

All patients in the EG achieved complete wound healing at last follow-up, compared to only 17 (68%) in the CG (*p* = 0.004). The likelihood of wound healing was 66% higher in the EG, although not reaching statistical significance (odds ratio (OR), 1.66 (95% confidence interval, CI, 0.80–3.44; *p* = 0.172)) (Table 3).

Bowel habit similarly improved in both groups from T2 to T4: the number of patients achieving normal stool consistency passed from 19 (76%) to 20 (80%) in the EG and from 18 (72%) to 22 (88%) in the CG (*p* = 0.846).

On last visit, all patients in both groups had returned to normal activities. A high level of satisfaction was reported by all patients (mean EG, 4.76 (0.52); mean CG, 4.60 (0.58)).

## 4. Discussion

Several medical and surgical strategies have been developed to improve the post-operative management of patients undergoing open excisional hemorrhoidectomy [4,16,17,18,19,20,21,22,23,24].

Post-operative pain is one of the oldest and most debated problems after hemorrhoidectomy. Although the VAS mean score was not statistically significantly lower over time in the EG vs. the CG, at POD 20 (T3), twenty patients in the former group achieved a higher benefit compared to the controls. A similar outcome was observed in a previous prospective multicenter study on the efficacy of mesoglycan in pain control after excisional hemorrhoidectomy [4]. The highest improvement achieved in the intervention group in post-operative pain symptoms at POD 20 was determinant for a faster return to work.

While burdened with higher recurrence rates, a better control of pain and faster recovery were observed after transanal hemorrhoidal dearterialization (THD) compared to open and closed excisional hemorrhoidectomies [16]. The absence of a surgical wound after THD may well explain these findings. On the other hand, patients totally unkeen to accept a risk of recurrence may reluctantly undergo THD.

Our results demonstrated that the use of Emorsan^®^Gel benefited all patients receiving the product in terms of wound healing at 40 days after surgery. While higher in the EG, the likelihood of wound healing did not reach statistical significance, possibly due to the small sample size. However, earlier wound healing observed in the EG may explain the better pain control at 20 days after surgery.

In a previous work, we highlighted the role of inflammation in the pathogenesis of HD by demonstrating a high level of matrix metalloproteinases in patients with III and IV degree hemorrhoids [25]. In this context, Emorsan^®^Gel forms a tight epidermal barrier and may reduce local oxidative stress through the action of polycarbophil and *Propionibacterium acnes* extract. Indeed, the radical scavenging property of *Propionibacterium* lysate prevents cell damage from oxygen free radicals, thus reducing the inflammatory process.

Such anti-inflammatory properties could contribute to limit post-operative edema that may eventually cause thrombosis. This often involves the muco-cutaneous bridges and represents the most frequent cause of pain. Of note, none of the patients in the EG experienced this complication, as opposed to 8% of subjects in the CG.

This study has some limitations including the non-randomization design and the small sample size. However, patients were recruited consecutively to mitigate the selection bias. We also acknowledge the lack of a standardized thrombosis-measuring tool.

## 5. Conclusions

The results of this study support the safety and effectiveness of Emorsan^®^Gel on pain control after open hemorrhoidectomy, promoting earlier wound healing compared to standard care treatment. Larger trials are needed to confirm such findings.

## Figures and Tables

**Table 1:** Patients’ characteristics.

	Emorsan^®^Gel *n* = 25	Controls *n* = 25	*p*-Value
Females (%)	10 (40)	12 (48)	0.776
Age (IQR)	51 (13)	48 (13)	0.899
Goligher grade			
III IV	12 13	13 14	0.898
ASA scoring system			
I II III	18 4 3	17 4 4	0.997
Ketorolac tromethamine (median daily dose in mg, IQR)			
POD 1 POD 2 POD 3 POD 4 POD 5	10 (12.5) 5 (10) 0 (5) 0 (0) 0 (0)	10 (12.5) 5 (7.5) 0 (5) 0 (0) 0 (0)	0.878 0.795 0.897 0.921 0.932

ASA: American Society of Anesthesiology; IQR: interquartile range; POD: post-operative day.

**Table 2:** Restricted maximum likelihood mixed model of the visual analogue scale (VAS) scores (between groups).

Δ VAS score	Coefficient	95% CI		*p*-Value
		Lower	Upper	
Emorsan^®^Gel	–0.09	–0.96	0.79	0.845
Age	0.01	–0.04	0.51	0.779
Gender	0.71	–0.81	0.95	0.874

CI: confidence interval.

**Table 3:** Mixed-effects logistic regression exploring the likelihood of wound healing controlling for age and gender.

Complete Wound Healing	OR	95% CI		*p*-Value
		Lower	Upper	
Emorsan^®^Gel	1.66	0.80	3.44	0.172
Age	0.99	0.96	1.03	0.798
Gender	1.53	0.73	3.24	0.262

OR: odds ratio; CI: confidence interval.
